# Osteoporosis in experimental postmenopausal polyarthritis: the relative contributions of estrogen deficiency and inflammation

**DOI:** 10.1186/ar1753

**Published:** 2005-04-27

**Authors:** Caroline Jochems, Ulrika Islander, Malin Erlandsson, Margareta Verdrengh, Claes Ohlsson, Hans Carlsten

**Affiliations:** 1 Department of Rheumatology and Inflammation Research at the Sahlgrenska Academy, Göteborg, Sweden; 2 Center for Bone Research at the Sahlgrenska Academy (CBS), Göteborg, Sweden

## Abstract

Generalized osteoporosis in postmenopausal rheumatoid arthritis (RA) is caused both by estrogen deficiency and by the inflammatory disease. The relative importance of each of these factors is unknown. The aim of this study was to establish a murine model of osteoporosis in postmenopausal RA, and to evaluate the relative importance and mechanisms of menopause and arthritis-related osteoporosis. To mimic postmenopausal RA, DBA/1 mice were ovariectomized, followed by the induction of type II collagen-induced arthritis. After the mice had been killed, paws were collected for histology, one femur for bone mineral density (BMD) and sera for analyses of markers of bone resorption (RatLaps; type I collagen cross-links, bone formation (osteocalcin) and cartilage destruction (cartilage oligomeric matrix protein), and for the evaluation of antigen-specific and innate immune responsiveness. Ovariectomized mice displayed more severe arthritis than sham-operated controls. At termination of the experiment, arthritic control mice and non-arthritic ovariectomized mice displayed trabecular bone losses of 26% and 22%, respectively. Ovariectomized mice with arthritis had as much as 58% decrease in trabecular BMD. Interestingly, cortical BMD was decreased by arthritis but was not affected by hormonal status. In addition, markers of bone resorption and cartilage destruction were increased in arthritic mice, whereas markers of bone formation were increased in ovariectomized mice. This study demonstrates that the loss of endogenous estrogen and inflammation contribute additively and equally to osteoporosis in experimental postmenopausal polyarthritis. Markers of bone remodeling and bone marrow lymphocyte phenotypes indicate different mechanisms for the development of osteoporosis caused by ovariectomy and arthritis in this model.

## Introduction

Rheumatoid arthritis (RA) is a common inflammatory joint disease with a prevalence of 0.5 to 1% [1]. RA is more common in women than in men, and the peak incidence in women coincides with the time of menopause [2]. There is evidence that the female sex hormone estrogen can influence both the incidence and the progression of RA. Exposure to oral contraceptives has been shown to reduce the risk of developing RA [3], and disease activity often decreases during pregnancy [4], when levels of female sex hormones are elevated. Recently, we reported beneficial effects of hormone replacement therapy in women with postmenopausal RA. Patients treated with hormone replacement therapy displayed increased bone mineral density (BMD), better clinical outcome, decreased erythrocyte sedimentation rate and elevated levels of serum hemoglobin as well as retarded progression of joint erosion [5].

RA is characterized by different skeletal manifestations including periarticular osteoporosis, bone erosions and generalized osteoporosis. The frequency of generalized osteoporosis in postmenopausal RA has been shown to be almost 50% [6,7], and these patients are at high risk for fractures. The bone loss in postmenopausal RA is believed to be caused by the combined effects of estrogen deficiency [8] and the inflammatory disease [9]. The relative importance of each of these two factors is not yet known.

Collagen-induced arthritis (CIA) is a well established murine model for human RA [10]. It has been shown that treatment with physiological doses of estradiol suppresses the disease progression in this model [11], whereas loss of endogenous estrogen by ovariectomy (OVX) leads to a more severe disease. OVX of mice leads to significant bone loss and is used as a model of postmenopausal osteopenia [12]. It has been demonstrated that OVX enhances the severity of arthritis and bone loss in CIA in rats, whereas exposure to estrogen suppresses it [13].

The aim of this study was to establish a murine model for studies of osteoporosis in postmenopausal RA, and to evaluate the relative importance and possible different mechanisms of estrogen deficiency versus joint inflammation for the induction of bone loss.

## Materials and methods

### Mice

The ethical committee for animal experiments at the University of Göteborg approved this study. Female DBA/1 mice (Taconic M&B A/S, Ry, Denmark) were kept, 5 to 10 animals to a cage, under standard environmental conditions and were fed with standard laboratory chow and tap water *ad libitum*.

### Castration

OVX or sham operation was performed at 10 weeks of age. Ovaries were removed by using a midline incision of the skin, and flank incisions of the peritoneum. The skin incision was closed with metallic clips. Sham-operated animals had their ovaries exposed but not removed. Surgery was performed under Ketalar^® ^(Pfizer AB, Täby, Sweden) and Domitor^® ^(Orion Pharma, Espoo, Finland) anesthesia.

### Induction and evaluation of arthritis

Nine days after surgery the mice were immunized with 100 μg of chicken type II collagen (CII; Sigma, St Louis, MO) dissolved in 0.1M acetic acid and emulsified with an equal volume of incomplete Freund's adjuvant (Sigma) supplemented with 0.5 mg/ml *Mycobacterium tuberculosis *(Sigma). A total volume of 100 μl was injected intradermally at the base of the tail (50 μl on each side). After 21 days mice received a booster injection in the same way using CII emulsified in incomplete Freund's adjuvant.

The animals were observed twice weekly for frequency and severity of arthritis. Severity was graded as described previously [14], scoring 1 to 3 in each paw (maximum of 12 points per mouse) as follows: 1, swelling or erythema in one joint; 2, swelling or erythema in two joints; 3, severe swelling of the entire paw or ankylosis.

### Tissue collection and histological examination

At 45 days after immunization mice were anaesthetized with Ketalar^®^/Domitor^®^, bled, and killed by cervical dislocation. Sera were individually stored at -20°C until use. Paws and femurs were collected.

Paws were placed in 4% paraformaldehyde dissolved in water, decalcified, and embedded in paraffin. Sections were stained with eosin/hematoxylin and encoded before examination. In each animal the front and back of all four paws were graded separately on a scale 0 to 4 and divided by 2, with a maximum of 16 points per mouse, as follows: 1, synovial hypertrophy; 2, pannus, erosions of cartilage; 3, erosions of bone; 4, complete ankylosis.

### Bone mineral density

One femur was subjected to a peripheral quantitative computed tomography (pQCT) scan with a Stratec pQCT XCT Research M, software version 5.4 B (Norland, Fort Atkinson, WI) at a resolution of 70 μm, as described previously [15]. Trabecular BMD was determined with a metaphyseal scan at a point 3% of the length of the femur from the growth plate. The inner 45% of the area was defined as the trabecular bone compartment. Cortical BMD was determined with a mid-diaphyseal scan, which contains only cortical bone.

### Serological markers of bone and cartilage remodeling

As a marker of bone resorption, serum levels of fragments of type I collagen were assessed using a RatLaps ELISA kit (Nordic Bioscience Diagnostics A/S, Herlev, Denmark). Serum levels of osteocalcin, a marker of bone formation, were determined with a Mouse Osteocalcin IRMA kit (Immutopics, Inc., San Clemente, CA).

As a marker of cartilage destruction, serum levels of COMP (cartilage oligomeric matrix protein) were determined with an Animal COMP^® ^ELISA kit (provided by AnaMar Medical AB, Uppsala, Sweden).

### Quantification of serum IgG and CII-specific antibodies

Serum levels of IgG were measured by single radial immunodiffusion as described previously [16]. By use of a previously described ELISA, serum levels of anti-CII antibodies were determined [17].

### Interleukin-6 bioassay

A bioassay [18] with cell line B13.29, subclone B9 (which is dependent on interleukin (IL)-6 for growth), was used to measure levels of IL-6 in serum. B9 cells were seeded with 5,000 cells per well into flat-bottomed 96-well plates (Nunc, Roskilde, Denmark) and cultured in Iscove's medium (Sigma) enriched with 50 μg/ml gentamicin (Sigma), 4 mM L-glutamine (Sigma), 50 μM mercaptoethanol (Sigma) and 10% fetal calf serum (Biological Ind., Beit Haemek, Israel). Sera were diluted 1:50 and added in triplicates. After 68 hours of culture, 1 μCi of ^3^H-thymidine (Amersham Pharmacia Biotech, Uppsala, Sweden) was added; the cells were harvested 4 hours later. Recombinant mouse IL-6 (National Institute for Biological Standards and Control, Potters Bar, Hertfordshire, UK) was used as a standard.

### Analysis of bone marrow cells

One femur was flushed with 2 ml of phosphate-buffered saline through the bone cavity to harvest bone marrow cells. After centrifugation at 515 *g *for 5 min, the pellet was resuspended in Tris-buffered 0.83% NH_4_Cl solution, pH 7.29, for 5 min to lyse erythrocytes, and then washed in phosphate-buffered saline. The cells were kept in complete Iscove's medium (described above) until use. Leukocytes were counted with an automated cell counter (Sysmex, Kobe, Japan).

The cells were stained with anti-CD45R/B220 conjugated with fluorescein isothiocyanate (clone RA3-6B2; BD) for B-lymphocytes and anti-CD3-conjugated with phycoerythrin (PE) (clone 145-2C11; BD), anti-CD4-biotin (clone RM4-5; BD), anti-CD8-biotin (clone 53-6.7; BD), anti-CD69-PE (clone H1.2F3; BD) and anti-CD25-PE (clone 7D4; BD) for T-lymphocytes. Cells were then subjected to fluorescence-activated cell sorting (FACS) analysis with FACSCalibur (BD Pharmingen, Franklin Lakes, NJ) and analyzed with Paint-A-Gate software (BD). Results are expressed as the numbers of positively stained cells per femur.

### Statistical analysis

For statistical evaluation the non-parametric Kruskal–Wallis test followed by a post hoc test was used between all four groups. A Mann–Whitney test was used when two groups were compared. *P *< 0.05 was considered statistically significant.

## Results

### OVX results in more severe arthritis

**Figure 1:**
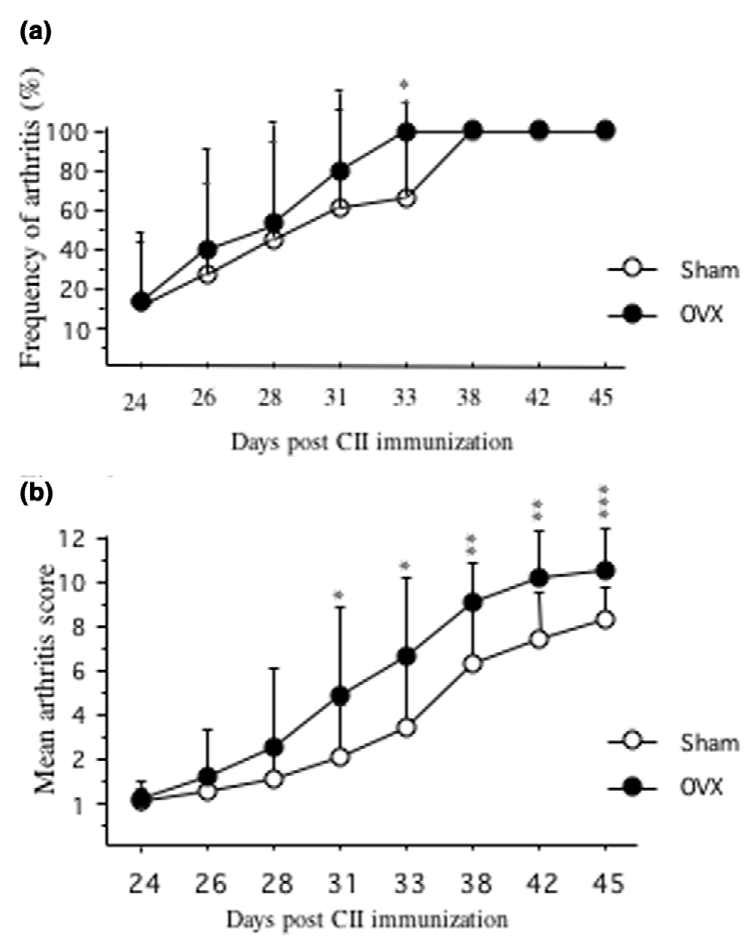
Mice after ovariectomy (OVX) displayed a significantly more severe disease than sham-operated mice. **(a) **The mice were observed twice weekly for frequency of arthritis. They were considered arthritic when they displayed signs of arthritis in one joint for two consecutive assessments, or arthritis in more than one joint. **(b) **Severity of arthritis was evaluated twice weekly. Severity was graded 1 to 3 in each paw (maximum 12 points per mouse). Open circles, sham (*n *= 18); filled circles, ovariectomy (*n *= 15). *P < 0.05; **P < 0.01; ***P < 0.001. CII, type II collagen.

Nine days after OVX/sham operation, mice were immunized (day 0) with chicken CII, and 3 weeks later (day 21) they received a booster injection. Arthritis developed from day 24, and arthritic score was evaluated twice a week. Ovariectomized mice displayed a more severe disease (Fig. 1) than sham-operated mice.

### Arthritis and loss of endogenous estrogen lead to an additive and similar degree of bone loss

**Figure 2:**
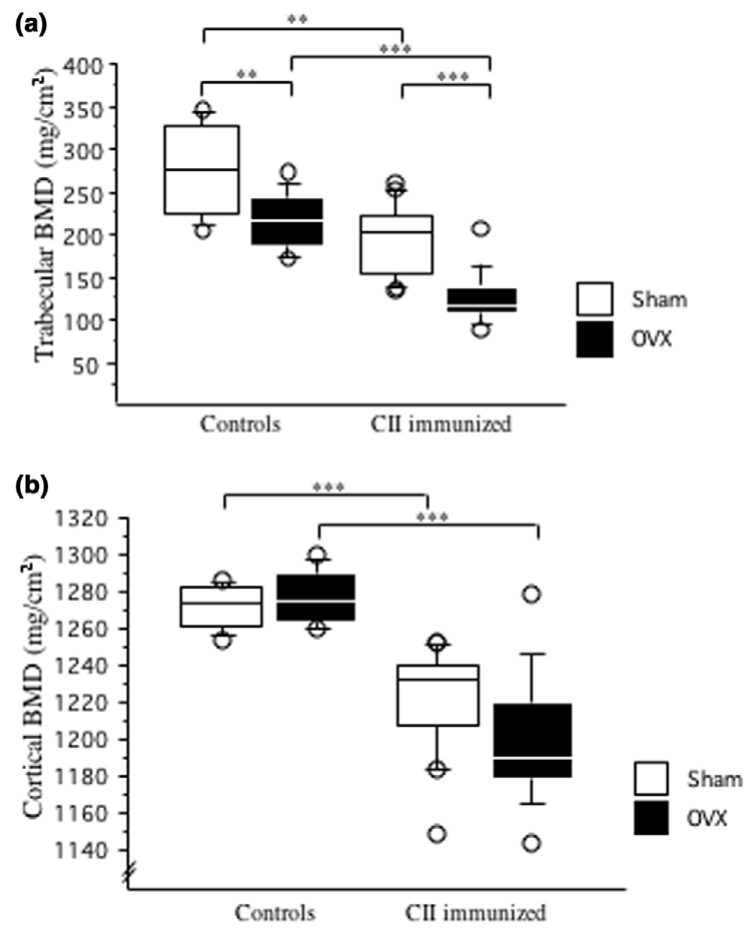
Ovariectomy decreased trabecular BMD whereas arthritis decreased both trabecular and cortical BMD. Peripheral quantitative computer tomography (pQCT) was performed to measure trabecular and cortical bone mineral density (BMD). **(a) **Trabecular bone mineral density (BMD) was determined with a metaphyseal scan at a point 3% of the length of the femur from the growth plate and the inner 45% of the area was defined as the trabecular bone compartment. **(b) **Cortical BMD of the femur was determined with a mid-diaphyseal scan. Results are shown as box plots (values are given as medians (horizontal lines), interquartile ranges (box) and ranges (whiskers); circles represent outliers). For controls, *n *= 10 for sham (open boxes) and ovariectomy (filled boxes); for immunized mice, *n *= 18 for sham and *n *= 14 for ovariectomy. **P < 0.01; ***P < 0.001. CII, type II collagen.

**Figure 3:**
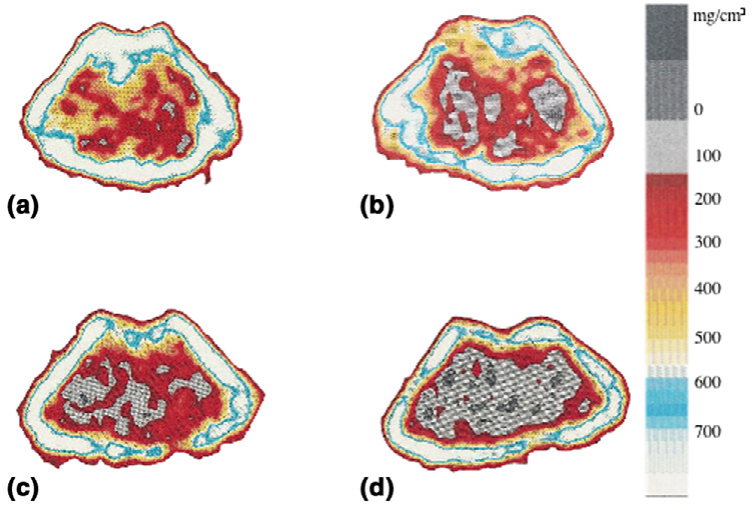
Peripheral quantitative computed tomography (pQCT) scans of one representative mouse in each group. Trabecular bone mineral density (BMD) was determined with a metaphyseal scan at a point 3% of the length of the femur from the growth plate and the inner 45% of the area was defined as the trabecular bone compartment. **(a) **Sham-operated control; **(b) **ovariectomy control; **(c) **sham-operated, arthritic mouse; **(d) **ovariectomized, arthritic mouse. The bar shows the density of the bone, from 0 (black) to 750 mg/cm^3 ^(white).

After termination of the experiment (day 45), BMD of the right femur was measured by pQCT. Mice subjected to OVX displayed a trabecular bone loss of 22% compared with sham-operated non-arthritic controls. Arthritic sham-operated mice displayed a bone loss of 26% and, finally, ovariectomized mice with arthritis had a 58% decrease in trabecular BMD (Figs 2a and 3). (These values were obtained by dividing the difference between the medians of each group and the sham-operated control group by the median of the sham-operated control group.) The cortical BMD was decreased by arthritis but was unaffected by hormonal status (Fig. 2b).

### Arthritis is associated with increased bone resorption, and OVX with increased bone formation

**Figure 4:**
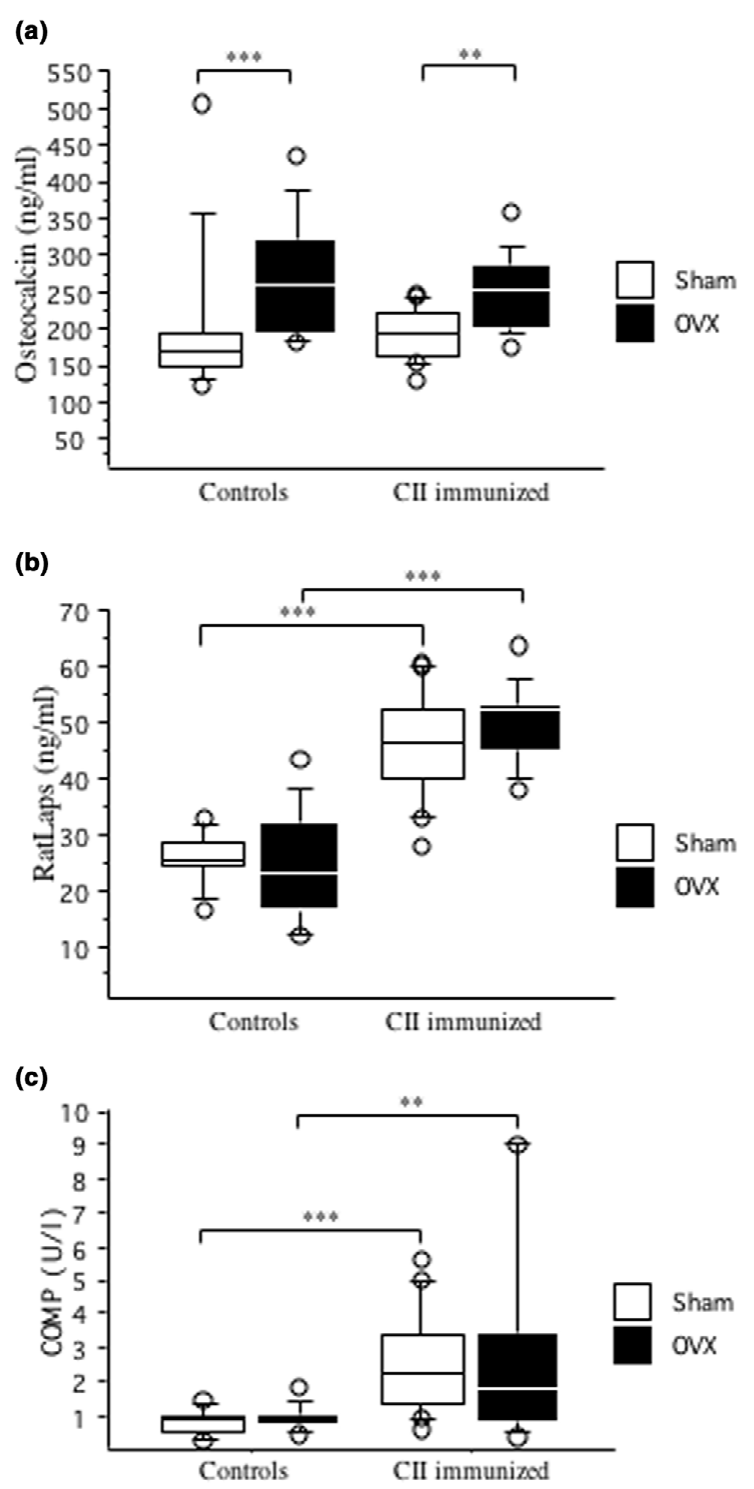
Ovariectomy increased bone formation and arthritis increased bone resorption and cartilage destruction. **(a) **Ovariectomy (OVX) increased bone formation. Serum levels of osteocalcin were analyzed by immunoradiometric assay. **(b) **Arthritis increased bone resorption. Serum levels of RatLaps were analyzed by ELISA. For controls, *n *= 10 for sham (open boxes) and ovariectomy (filled boxes); for immunized mice, *n *= 18 for sham and *n *= 15 for ovariectomy. **(c) **Arthritis increased cartilage destruction. Serum levels of cartilage oligomeric matrix protein (COMP) were analyzed by ELISA. For controls, *n *= 9 for sham (open boxes) and *n *= 10 for ovariectomy (filled boxes); for immunized mice, *n *= 17 for sham and *n *= 14 for ovariectomy. **P < 0.01, ***P < 0.001. Results are shown as box plots (values are given as medians (horizontal lines), interquartile ranges (box) and ranges (whiskers), circles represent outliers).

At day 45, serum levels of osteocalcin were increased in ovariectomized mice compared with sham-operated mice (Fig. 4a). Immunization with CII did not affect the levels of osteocalcin. Serum levels of RatLaps (type I collagen cross-links) were greatly enhanced in the CII-immunized mice, in comparison with controls (Fig. 4b). In contrast, OVX did not increase the levels of RatLaps.

### Arthritis, but not estrogen deficiency, increases cartilage destruction

Serum levels of COMP were increased in arthritic mice but were not affected by hormonal status (Fig. 4c).

### Hormonal status does not affect arthritis-induced increased levels of pro-inflammatory cytokines, IgG and CII antibodies

As shown in Table 1, serum levels of the pro-inflammatory cytokine IL-6 were low in non-arthritic mice in comparison with the higher levels found in arthritic mice. All arthritic mice displayed high serum levels of IgG and anti-CII antibodies, but no significant differences between the ovariectomized and sham-operated mice were demonstrated.

### Phenotypes of bone marrow lymphocytes are influenced both by OVX and by arthritis

Flow cytometry analysis was performed to evaluate the effects of OVX and arthritis on phenotypes of bone marrow lymphocytes (Table 2). OVX was associated with an increased number of B lymphocytes per femur, whereas CII immunization led to a decreased number of B cells. The total numbers of T lymphocytes (CD3^+^) and CD4^+ ^cells per femur were not affected by either OVX or CII immunization. In contrast, the number of CD8^+ ^cells was significantly decreased in both sham-operated and ovariectomized arthritic mice compared with controls. The CD69 expression, a marker of early activation, was increased on CD4^+ ^and CD8^+ ^cells in arthritic mice. In contrast, T cell CD25 expression remained unchanged in all groups (data not shown).

### Histological findings

There was no significant difference in the degree of histological destruction score between ovariectomized and sham-operated arthritic mice (Table 1).

## Discussion

Osteoporosis is one of the major problems in postmenopausal RA [7,19] and is a factor contributing to increased risk for fractures [20]. The mechanisms and relative importance of estrogen deficiency versus inflammation for the bone loss in postmenopausal RA are not fully understood. Our study is the first to demonstrate equal contributions of estrogen deficiency and polyarthritis to bone loss in a model of human postmenopausal RA. In addition, serum markers of bone and cartilage turnover and FACS analysis of bone marrow leukocyte phenotypes indicate different mechanisms for the development of osteoporosis.

OVX of the DBA/1 mice several weeks before the development of arthritis enabled separate and concurrent analyses of the effects of estrogen deficiency and the inflammatory disease on bone loss. Our results show that the loss of endogenous estrogen and the ongoing arthritic disease cause a similar degree of trabecular bone loss (22% and 26%, respectively) and clearly have an additive effect, because ovariectomized mice with arthritis lost 58% of trabecular BMD. Interestingly, arthritis also induced a significant decrease in cortical BMD, whereas OVX, irrespective of inflammatory status, did not affect this parameter.

It has previously been demonstrated in CIA in rats that OVX enhances the severity of arthritis and bone loss, whereas exposure to estrogen suppresses it [13]. A more detailed comparison between the previous study and ours is not possible because we ovariectomized the mice 2 weeks before initial immunization (that is, 5 weeks before the development of arthritis) to achieve an established postmenopausal state, whereas Yamasaki and colleagues ovariectomized the rats 1 week after sensitization.

Systemic inflammation, impaired physical activity, low body mass and treatment with corticosteroids are some important factors associated with the development of osteoporosis in RA. The pathophysiological mechanisms of bone loss in arthritis have been shown to be mediated through the activation of osteoclasts by the macrophage-derived proinflammatory cytokines tumor necrosis factor-α and IL-1, and by the production of RANKL by activated T-lymphocytes and fibroblasts. Garnero and colleagues [21] found increased serum levels of markers of bone resorption in patients with erosive RA, and decreased markers of bone formation. The discrepancy between bone formation and bone resorption results in the enhanced bone loss in arthritis.

We showed that there was strongly increased bone resorption measured by RatLaps in the arthritic mice but not in ovariectomized mice. This was expected, as we sought to study changes in established menopause, and not the rapid phase of bone loss that follows OVX. In contrast to what Garnero and colleagues found in RA patients, we failed to demonstrate decreased serum levels of osteocalcin associated with arthritis. In accord with our results, Nishida and colleagues [22] have previously suggested that reduced bone formation might not be a substantial contributor to bone loss in DBA/1 mice, so this difference might be species dependent.

The exact mechanism whereby OVX induces bone loss in mice is not yet known. Several mechanisms are involved, and recent studies have shown that OVX of mice was associated with an increase in the number of activated, tumor necrosis factor-producing, bone marrow T lymphocytes stimulating monocytes to differentiate into osteoclasts [12,23,24]. We did not show an increase in bone marrow T lymphocytes. The explanation for this discrepancy could be either that we used CD3 as a marker for T cells, whereas others have used anti-CD90 (which is also expressed on natural killer cells, monocytes and dendritic cells), or the very late time point (8 weeks after OVX) that we used for analysis of the bone marrow. Indeed, the finding that RatLaps, a serum marker of bone resorption, was unaltered whereas osteocalcin, a serum marker of bone formation, was increased in ovariectomized mice indicates that the period of OVX-induced increased activation of osteoclasts had already ended at this late time point. As has been shown previously, the number of B lymphocytes in bone marrow was increased after OVX [25] and decreased in the arthritic mice [26]. As increased B lymphopoiesis has been shown to be associated with bone loss [27], our data suggest separate mechanisms for the bone loss found in estrogen deficiency and in arthritis.

COMP is an extracellular matrix protein initially found in cartilage but recently also shown to be secreted by synovial fibroblasts. Serum levels of COMP are used as a marker of cartilage destruction and have previously been evaluated in CIA in rats [28,29]. We found increased serum COMP levels in all arthritic mice, irrespective of the estrogen level, indicating a lack of cartilage protection by endogenous ovarian hormones.

Taken together, although the analyses in this study were all performed on day 45, the differences in serum levels of RatLaps, osteocalcin, COMP and frequencies and phenotypes of bone marrow lymphocytes between mice subjected to OVX and CIA suggest the possibility of different mechanisms for the development of osteoporosis in estrogen deficiency and arthritic disease.

The female sex hormone estradiol not only preserves bone but also has a clear anti-arthritic effect both in human RA [4,5] as well as in rat [13] and murine [11,30] CIA. Clinically, the arthritic ovariectomized mice developed a more severe disease than the sham-operated mice. However, at termination of the experiment all mice, irrespective of hormonal status, had developed severe arthritic disease, with histological destruction score, pro-inflammatory cytokines and CII antibodies at similar levels.

## Conclusion

We demonstrate that CIA in ovariectomized DBA/1 mice is a relevant model for studies of osteoporosis in postmenopausal RA. Furthermore, the loss of endogenous estrogen and the inflammation contribute equally to bone loss in this model. Markers of bone and cartilage turnover, as well as bone marrow lymphocyte phenotypes, indicate different mechanisms for bone loss induced by estrogen deficiency and inflammation, respectively. We suggest that this model is well suited for future studies, both on anti-arthritic and anti-osteoporotic properties of new medications and on mechanisms for bone loss in postmenopausal polyarthritis.

## Abbreviations

BMD = bone mineral density; CIA = collagen-induced arthritis; CII = type II collagen; COMP = cartilage oligomeric matrix protein; ELISA = enzyme-linked immunosorbent assay; FACS = fluorescence-activated cell sorting; IL = interleukin; OVX = ovariectomy; pQCT = peripheral quantitative computed tomography; RA = rheumatoid arthritis.

## Competing interests

The author(s) declare that they have no competing interests.

## Authors' contributions

HC and CO participated in study design, interpretation of data and manuscript preparation. UI aided with analysis of data and statistical analysis. ME and MV aided with acquisition of data. The study was performed mainly by CJ. All authors read and approved the final manuscript.

## Figures and Tables

**Table 1:** Serological markers of inflammation and histopathological findings were not significantly affected by ovariectomy

Arthritis	OVX	No. of mice	IgG (mg/ml)	CII antibody (ng/ml)	Interleukin-6 (pg/ml)	Frequency of arthritis, day 45 (%)	Arthritic score, day 45	Histopathology (score)
-	-	10	10 (10–12)	n.d	62 (48–67)	0	0	0
	+	10	10 (7–15)	n.d	80 (42–111)	0	0	0
+	-	18	18 (15–23)	4.6 (2.6–8.0)	343 (214–755)	100	8 (7–10)***	9.5 (7.0–11.5)
	+	15	18 (15–18)	3.5 (2.3–4.6)	371 (280–602)	100	11 (10–12)	11.0 (8.9–13.0)

Values are medians and interquartile ranges for each group. The maximum arthritic score was 12 points per mouse. ***P < 0.001 between sham-operated and ovariectomized arthritic mice. CII, type II collagen; n.d., not detectable; OVX, ovariectomy.

**Table 2:** Characteristics of bone marrow lymphocytes were influenced both by ovariectomy and by arthritis

Arthritis	OVX	*n*	Bone marrow cellularity (× 10^6^)	B cells per femur (× 10^6^)	T cells per femur (× 10^6^)	CD4^+ ^cells per femur (× 10^6^)	CD69^+^/CD4^+ ^cells (%)	CD8^+ ^cells per femur (× 10^6^)	CD69^+^/CD8^+ ^cells (%)
-	-	10	5.1 (4.0–6.4)	1.5 (1.0–1.7)***	0.06 (0.05–0.09)	0.02 (0.02–0.04)	25 (18–30)	0.013 (0.008–0.018)	3 (1–3)
	+	10	6.0 (5.0–8.6)	2.4 (2.0–3.0)	0.05 (0.04–0.07)	0.02 (0.02–0.03)	29 (26–31)	0.008 (0.006–0.012)	4 (2–6)
+	-	18	5.2 (4.7–7.0)	1.0 (0.8–1.3)**^†^	0.05 (0.04–0.06)	0.02 (0.01–0.02)	50 (43–58)***^†††^	0.004 (0.002–0.007)^†††^	9 (4–25)^†††^
	+	15	6.1 (5.3–8.4)	1.5 (1.0–2.5)^†^	0.05 (0.03–0.05)	0.02 (0.01–0.02)	35 (29–40)	0.005 (0.002–0.006)^†††^	10 (5–16)^†^

Values are medians and interquartile ranges for each group; *n *is the number of mice. Comparison between sham operation and ovariectomy (OVX): **P < 0.01; ***P < 0.001. Comparison between arthritic mice and their controls: ^†^*P *< 0.05; ^†††^*P *< 0.001.
